# *MAPKAP1* rs10118570 Polymorphism Is Associated with Anti-Infection and Anti-Hepatic Fibrogenesis in Schistosomiasis Japonica

**DOI:** 10.1371/journal.pone.0105995

**Published:** 2014-08-25

**Authors:** Xiao Zhu, Jinfang Zhang, Wenguo Fan, Yunguo Gong, Jianhua Yan, Zhidong Yuan, Lang Wu, Hongjing Cui, Haiqing Luo, Qingming Kong, Li Tang, Shuilong Leng, Yufeng Liao, Weiming Fu, Qin Xiao, Dongpei Li

**Affiliations:** 1 Guangdong Province Key Laboratory of Medical Molecular Diagnosis, Department of Clinical Oncology, Guangdong Medical College, Zhanjiang/Dongguan, China; 2 Peking University Shenzhen Hospital, Shenzhen Peking University - The Hong Kong University of Science and Technology Medical Center, Shenzhen, China; 3 Department of Orthopaedics & Traumatology, The Chinese University of Hong Kong, Prince of Wales Hospital, Hong Kong, China; 4 Guangdong Provincial Key Laboratory of Stomatology, Guanghua School of Stomatology, Sun Yat-sen University, Guangzhou, China; 5 School of Biomedical Informatics, University of Texas, Houston, Texas, United States of America; 6 Clinical Imaging Research Center, A*STAR-NUS, Singapore, Singapore; 7 School of Life Sciences, Hunan University of Science and Technology, Xiangtan, China; 8 Center for Clinical and Translational Science, Mayo Clinic, Rochester, Minnesota, United States of America; 9 Immunity and Biochemical Research Lab, Zhejiang Academy of Medical Sciences, Hangzhou, China; 10 Schistosomiasis Institute, Hubei Academy of Preventive Medicine, Hubei Provincial Center for Disease Control and Prevention, Wuhan, China; 11 Department of Human Anatomy, Guangzhou Medical University, Guangzhou, China; 12 Department of Laboratory Medicine, Ningbo No.2 Hospital, Ningbo, China; 13 Guangzhou Institute of Advanced Technology, Chinese Academy of Sciences, Guangzhou, China; 14 Institute of Molecular Medicine and Genetics, Department of Neuroscience and Regenerative Medicine, Georgia Regents University, Augusta, Georgia, United States of America; Lady Davis Institute for Medical Research/McGill University, Canada

## Abstract

Chronic infection with *Schistosoma japonicum* is an important cause of hepatic fibrosis (HF). Human 9q33.3 is one of the most important loci for stress-related diseases. We examined the potential associations of 43 single-nucleotide polymorphisms (SNPs) with *S. japonicum* infection and HF in epidemic region in China. We identified a SNP (rs10118570 GG in *mitogen-activated protein kinase associated protein 1*, *MAPKAP1*) contributes to anti-infection (adjusted OR = 0.35) and anti-fibrogenesis (adjusted RR = 0.44) in the discovery study. Replicative and combined studies showed consistent protective quality for this genotype (replicative: adjusted OR = 0.37 for anti-infection, and adjusted RR = 0.40 for anti-fibrogenesis; Combined: adjusted OR = 0.45 for anti-infection, and adjusted RR = 0.42 for anti-fibrogenesis). Univariate and multivariate analysis in the discovery, replicative and combined studies, suggested that durations (years), splenomegaly, serum ALB and rs10118570 were independent predictors influencing the fibrogenesis. The analysis of gene-gene interaction showed rs10118570 functions independently. We conclude that *MAPKAP1* may represent a novel anti-infection and anti-fibrogenesis genomic locus in chronic schistosomiasis japonica. And rs10118570 may be a potential biomarker and target for the treatment of this life-threatening ancient disease.

## Introduction

Schistosomiasis remains one of the most prevalent parasitic infections in the world. It is endemic in 76 countries and territories, and continues to be a global public health concern in the developing world [1], [2]. Schistosomiasis japonica, caused by *Schistosoma japonicum*, has been endemic in China since ancient times. It is one of the five species of schistosomes that infected about 800,000 Chinese people [3], [4], [5]. Schistosomiasis japonica is one of many zoonotic parasitic diseases along the Yangtze River and in the south of China, and remains a major public health problem in China today.

As far as is known live ripe worms have little or no pathogenic effect on the mammalian host and chronic dysfunction is principally owing to the deposition of eggs in the hepatic and intestinal tissues, with inflammation, granuloma formation and complicated fibrosis [6]. The adult worms do not replicate within the definitive host and the extent of the disorder depends largely on the number of eggs that are reserved in the tissues, which in turn depends on the number, egg-laying capability and longevity of adult worms within the host [7], [8]. Therefore, host genetic factors might play an important role in determining the extent of symptom ensuing from infection. In fact, some studies have revealed certain mutations in genome (such as Ficolin-2, mannose-binding lectin *et al.*) may be associated with protection or susceptibility to schistosomiasis [9], [10], [11].

The *Homo sapiens* 9q33.3 region is an important locus for several diseases with a complex genetic background. Recent candidate gene approaches have reported single-nucleotide polymorphisms (SNPs) in this region to be associated with several important diseases including HBV related-cirrhosis [12], [13], [14], hepatocellular carcinoma (HCC) [15], [16], [17], [18], lung cancer [19], [20], [21], gastric and colorectal cancer [22]. These findings let us to undertake the case-control studies to identify genes for chronic schistosomiasis on 9q33.3.

## Materials and Methods

### Ethics statement

The study protocol was approved by the Ethics Committee of Guangdong Medical College and adhered to the tenets of the Declaration of Helsinki. Additionally, written informed consent was obtained from each participant.

### Study area and population

From October 2006 to December 2010, the chronic schistosomiasis patients in this study were selected from Jiangling County (along the Yangtz River and near the Dongting River) and Yangxin County (along the tributary of the Yangtz River and near the Poyang River) in China, where are highly endemic for *S. japonicum*. 525 cases and 532 healthy controls with medical examination were recruited as the discovery cohort from Yangxin Center for Disease Control and Prevention and Yangxin County Hospital. The replicative cohort consisted of 489 cases and 1247 healthy controls from Jiangling Center for Disease Control and Prevention and Jiangling County Hospital. Both of the discovery cohort and the replicative cohort were included in the combined study.

All individuals were interviewed with a questionnaire with respect to their age, gender, occupation, water contact, schistosomiasis history, previous praziquantel treatment as part of a government control program. All controls and cases had similar water exposure at risk for exposure to S. japonicum. All cases were selected from the population of farmers or fishermen who had suffered from Schistosomiasis for ≥15 years and had regular water contact. Individuals susceptible to chronic infection were defined as those who had been diagnosed (by serologic and ultrasonographic examinations, and fecal egg count) with a *S. japonicum* infection that was accompanied by three of the following symptoms: low fever, loss of appetite, weakness, headaches and dizziness. Individual infection with *S. japonicum* was assessed by both semi-quantitative Kato-Katz thick smear stool examination and the miracidium hatching test, a traditional method developed in China [23], [24]. The data of clinical and laboratory were collected on the date diagnostic liver imaging was executed. A complete physical examination and medical history was performed in all cases. Body mass index (BMI) was defined as the individual’s body mass divided by the square of her/his height. Smokers were defined by valuing subjects who have smoked more than an average of 2 cigarettes per day or who have smoked more than 1000 cigarettes over a lifetime. If a participant consumed mean 59 grams of alcohol per week it means that he or she is an alcohol drinker. Laboratory evaluation included hemoglobin level and blood cell count (corpuscular volume, white blood cell and eosinophile granulocyte count); serology for tubercle bacillus, hepatitis B and C infection; serum alpha-fetal protein (AFP) level; and routine liver biochemistry (total bilirubin, albumin and alanine transaminase (ALT) levels. All cases had received free standardized praziquantel treatment partly financed by the World Health Organization (WHO) grants in the local service systems. The control series were healthy adults after physical checkup. All participants were exclude tuberculosis and/or HCV infection and cancer history. The main features of the subjects included are summarized in Table 1.

**Table 1:** Clinical and laboratory features of the subjects included in the study*.

Characteristics	Discovery cohort			Replication cohort			Combined cohort		
	Cases		Controls	Cases		Controls	Cases		Controls
	Non-HF	HF		Non-HF	HF		Non-HF	HF	
Sample size	148	377	532	147	342	1247	295	719	1779
Age (ys)									
Mean ± SD	43.5±9.8	47.2±6.2	33.0±9.2	47.9±7.7	41.3±8.4	35.6±12.3	45.9±10.1	44.6±8.3	34.8±11.1
Gender (%)									
Female	87 (58.78)	45 (11.94)	173 (32.52)	64 (43.54)	51 (14.91)	543 (43.54)	151 (51.19)	96 (13.35)	716 (40.25)
Male	61 (41.22)	332 (88.06)	359 (67.48)	83 (56.46)	291 (85.09)	704 (56.46)	144 (48.81)	623 (86.65)	1063 (59.75)
BMI, kg/m2, (±SD)									
Female	21.2±2.0	20.4±1.9	21.9±2.1	22.7±1.8	21.4±2.3	22.2±2.2	21.9±1.9	20.9±2.2	22.1±2.2
Male	22.5±1.9	19.0±2.1	22.0±2.1	21.3±2.6	20.8±2.0	22.7±2.3	21.8±2.4	19.7±2.0	22.5±2.3
Smoking (n, %)	45 (30.41)	204 (54.11)	115 (21.62)	39 (26.53)	118 (34.50)	357 (28.63)	84 (28.47)	322 (44.78)	472 (26.53)
Drinking (n, %)	23 (15.54)	115 (30.50)	147 (27.63)	27 (18.37)	109 (31.87)	246 (19.73)	50 (16.95)	224 (31.15)	393 (22.09)
Durations schistosomiasis (ys, mean ± SD)	27.3±14.8	25.6±13.5	n/a	26.7±14.6	28.5±16.4	n/a	27.0±14.7	27.1±15.9	n/a
Fibrosis grade (%)									
0	148 (100)	0 (0.00)	532 (100)	147 (100)	0 (0.00)	1247 (100)	295 (100)	0 (0.00)	1779 (100)
I	0 (0)	161 (42.71)	0 (0)	0 (0)	127 (37.13)	0 (0)	0 (0)	288 (40.06)	0 (0)
II	0 (0)	126 (33.42)	0 (0)	0 (0)	129 (37.72)	0 (0)	0 (0)	255 (35.47)	0 (0)
III	0 (0)	71 (18.83)	0 (0)	0 (0)	58 (16.96)	0 (0)	0 (0)	129 (17.94)	0 (0)
IV	0 (0)	19 (5.04)	0 (0)	0 (0)	28 (8.19)	0 (0)	0 (0)	47 (6.54)	0 (0)
Hepatomegaly (%)†	14 (9.46)	101 (26.79)	0 (0)	11 (7.48)	115 (33.63)	0 (0)	25 (8.47)	216 (30.04)	0 (0)
Splenomegaly (%)‡	4 (2.70)	47 (12.47)	0 (0)	3 (2.04)	38 (11.11)	0 (0)	7 (2.37)	85 (11.82)	0 (0)
Hemoglobin level g/dL, mean (±SD)	12.4±2.7	11.1±2.5	12.3±2.4	12.1±2.6	11.5±2.8	12.5±2.5	12.2±2.7	11.4±2.7	12.5±2.4
Corpuscular volume, mean (fL, ±SD)	89.5±7.0	93.2±9.8	88.7±8.5	90.7±7.5	91.2±10.1	89.5±8.1	89.9±7.4	92.3±9.9	89.1±8.3
WBC, mean(×10^9^/L, ±SD)	5.9±2.0	4.9±1.7	7.3±2.3	6.7±2.4	5.8±3.0	7.5±2.4	6.3±2.2	5.4±2.6	7.4±2.4
Eosinophile granulocyte, mean (×10^9^/L, ±SD)	0.30±0.09	0.46±0.06	No data	0.27±0.07	0.47±0.10	No data	0.28±0.08	0.46±0.08	No data
HBV (HBsAg +, %)	7 (4.73)	24 (6.37)	0 (0)	13 (8.84)	29 (8.48)	0 (0)	20 (6.78)	53 (7.37)	0 (0)
Anti-HCV	0 (0)	0 (0)	0 (0)	0 (0)	0 (0)	0 (0)	0 (0)	0 (0)	0 (0)
Anti- tubercle bacillus	0 (0)	0 (0)	0 (0)	0 (0)	0 (0)	0 (0)	0 (0)	0 (0)	0 (0)
Serum AFP (n, %)									
<25 ng/ml	146 (98.65)	308 (81.70)	532 (100)	144 (97.96)	284 (83.04)	1247 (100)	290 (98.31)	592 (82.34)	1779 (100)
≥25 ng/ml	2 (1.35)	69 (18.30)	0 (0)	3 (2.04)	58 (16.96)	0 (0)	5 (1.69)	127 (17.66)	0 (0)
Liver function (mean ± SD)									
T-Bil (µmol/L)	14.1±9.5	28.6±20.9	11.8±6.9	17.5±8.2	30.0±19.0	11.2±7.2	15.9±9.2	29.2±20.5	11.6±7.2
ALB (g/L)	41.1±6.8	35.6±7.4	43.5±7.2	44.5±7.0	34.9±6.6	43.8±7.6	42.7±6.9	35.3±7.2	43.6±7.5
ALT (IU/L)	30.6±11.3	45.0±22.7	21.8±13.7	27.2±12.9	39.0±19.9	23.3±8.5	28.6±12.5	42.1±21.9	22.6±13.4

*HF, hepatic fibrosis; SD, standard deviation; BMI, body mass index; WBC, white blood cell; HBV, hepatitis B virus; AFP, alpha-fetal protein; T-Bil, total bilirubin; ALB, albumin; ALT, alanine transaminase.

† Length of left liver lobe, a longitudinal section at left parasternal line. Length of right liver lobe, anterior axillary view and maximum oblique diameter between front and back sections with inspiration.

‡ Thickness of spleen from hilum to opposite section. Length of spleen in left oblique view with maximum length in a section through the splenic hilus.

### Diagnosis of hepatic fibrosis (HF)

Fibrosis or cirrhosis is a diagnosis and is often confused with portal hypertension, which results from fibrosis/cirrhosis [25]. Reliable diagnosis of fibrosis or cirrhosis usually counts on liver biopsy. Percutaneous biopsy is a dependable handling when operated by experienced doctors; however, patients with hepatopathy and other bleeding diatheses can often have an incremental hemorrhagic risk requiring treatment. Imaging techniques may display an atrophic liver or evidence of portal hypertension but do not allow visualization of fibrosis or cirrhosis. However, the World Health Organization (WHO) still recommended non-invasive techniques instead of biopsy in schistosomiasis endemic regions in developing countries [26].

Ultrasonography is a useful tool for morbidity assessment of HF in developing countries, which was widespreadly used in rural health clinics in China, partly supported by the Special Programmes of World Bank. However, in order to make full use of this technique, the application in local schistosomiasis endemic regions needs to be preferential developed. All controls and patients in our study underwent extensive ultrasonographic examination. The diagnosis of HF was based on the WHO guidelines modified as indicated for *S. japonicum* infections, although the WHO scale grades parenchymal (ParF) and periportal fibrosis (PPF) separately [26]. The grade is specified a number in view of the extent degree of immunoinflammation, which is usually scored from 0∼IV with 0 being no fibrosis and IV considered cirrhosis. Briefly, the ultrasonography diagnosis criteria of HF in schistosomiasis endemic regions in China were summarized in Table 2.

**Table 2:** The ultrasonography diagnosis criteria of hepatic fibrosis in schistosomiasis endemic regions in China.

Grade	Diagnosis criteria
Grade 0	No scarring and absence of fibrosis.
Grade I	Minimal scarring or fibrous expansion in some perisinusoidal or portal areas; an uninterrupted U-shaped echogenic band usually greater than 4 mm in diameter extended from the left portal vein bifurcation branch to the gallbladder bed.
Grade II	Fibrous expansion and scarring in most perisinusoidal and portal/periportal, with occasional portal-to-portal bridging; an echogenic band with a diameter greater than 10 mm surrounding the central part and major branches of the portal vein, extended external surface of the liver that contains blood vessels.
Grade III	Streak-like septal and fibrous bands of portal areas with conspicuous bridging including portal-to-portal and portal-to-central bridging, spreading into portal vein lumina and periphery of the liver.
Grade IV	Advanced scarring or cirrhosis of the liver, usually a liver lobule completely surrounded by scarring.

### SNP selection

To identify Schistosomiasis susceptibility genes on *Homo sapiens* chromosome 9q33.3, we selected haplotype tagging single-nucleotide polymorphism (htSNPs) from the International HapMap Project database as well as random SNPs from the National Center for Biotechnology Information SNP Database, Ensembl database, and GWAS Central. The selected SNPs were high heterozygosity with minor allele frequencies >10%, tagging of the most common haplotypes, and coverage of the main blocks of linkage disequilibrium (LD) in the Han Chinese population at approximate intervals of 64 kb across the euchromatic regions of chromosome 9q33.3. We selected 45 SNPs spanning *GRP78* and its flanking genes (∼3.0 Mb) that met these selection criteria and had previously shown robust genotyping efficacy and important functions.

### Genotyping

Genomic DNA was extracted from peripheral blood leukocytes using QIAGEN QIAamp DNA Mini Blood Kit (Hilden, Germany). DNA sample was collected in a 1.5 ml Eppendorf tube, stored at −20°C until use. The GeneBank accession number used in this study was NT_008470.18. PCRs were performed in a 50 µl reaction systems containing 200 ng sample DNA, 5 µl of 10× *Ex Taq* buffer (Mg^2+^ free; Takara, Japan), 2 mM MgCl_2_, 20 pmol of each primer, 0.2 mM of each deoxynucleoside triphosphates (dNTPs), and 5U *Ex Taq* polymerase (Takara, Shiga, Japan). PCR products were extracted from the gels using the QIAquick PCR purification kit (QIAGEN, CA, USA) for resequencing. Resequencing was performed directly with one of the PCR primers or other specified primers, Taq polymerase, ABI PRISM BigDye terminators on an ABI 3730xl DNA Analyzer (Applied Biosystems, Inc., Foster City, CA, USA). The primers for PCR and re-sequencing are available on request.

### Statistical analysis

The associations of the variant SNPs with *S. japonicum* incidence were evaluated in logistical regression. Factors that might affect the development of HF, i.e., age, gender, smoking and drinking habits were included as covariates in the adjusted relative risk analyses.

In multiple comparisons, the experimentwise significance (*P*<1.163×10^−3^) was determined by Bonferroni correction based on the total number of markers genotyped. The Cochran - Armitage Trend Test was performed with Plink v1.07 to examine the trend between variant carriers and case patients with *S. japonicum*. The Hardy-Weinberg equilibrium (HWE) was tested using Chi-square test. The *P* value for HWE >1.163×10^−3^ was considered to conform to population genetic law.

In order to account for multiple testing, empirical values to correct for the occurrence of false positives were ascertained by using the max(T) permutation procedure with 10^5^ permutations for each tested marker. We used PLINK v1.07 to calculate the overall *P* values for the SNPs in the Chinese population using 10^5^ permutations, to perform the case-control association tests for single SNPs, to model the genotypic associations. Empirical *P*-values (*P*_empirical_)<0.05 were considered significant. The quantile-quantile (Q-Q) plot was constructed using the R-package script.

Single- and multilocus analyses were performed using Haploview version 4.0. Based on the linkage disequilibrium (LD) structure, haplotype blocks were inferred under the default algorithm of Gabriel et al. [27] and displayed schematically with interblock connections. To characterize the LD pattern, we calculated pairwise D’ and r^2^ values using two-SNP haplotypes inferred via the EH plus program [28]. The effective number of haplotypes was calculated as n_e_ = 1/∑p_i_^2^, where p_i_ are the individual haplotype frequency estimates [29].

Relative risk (RR) was calculated with multivariate regression according to the ad rationem of HF in patients with chronic schistosomiasis. We appraised various factors that might influence the development of fibrosis and have included them as covariates in the multivariate analysis: age, gender, smoking and drinking habits, HBV infection, durations of disease in the past years.

The appointed terminus of fibrosis was the presence or absence of advanced grade fibrosis (III, IV). Univariate descriptive statistic was used to compare patients with and without advanced HF. Each factor was included in a multivariate Cox regression analysis with hazard ratios (HR) and 95% confidence intervals (CIs) to appraise variables independently associated with presence or absence of advanced HF. Duration of schistosomiasis was from the first attack to the time of blood sample collection according to schistosomiasis history in questionnaire.

The gene-gene interaction in this study means SNP-SNP interaction, which was carried out by Chi-square test.

## Results

### 9q33.3 and *S. japonicum* infectious risk in the discovery study

**Figure 1:**
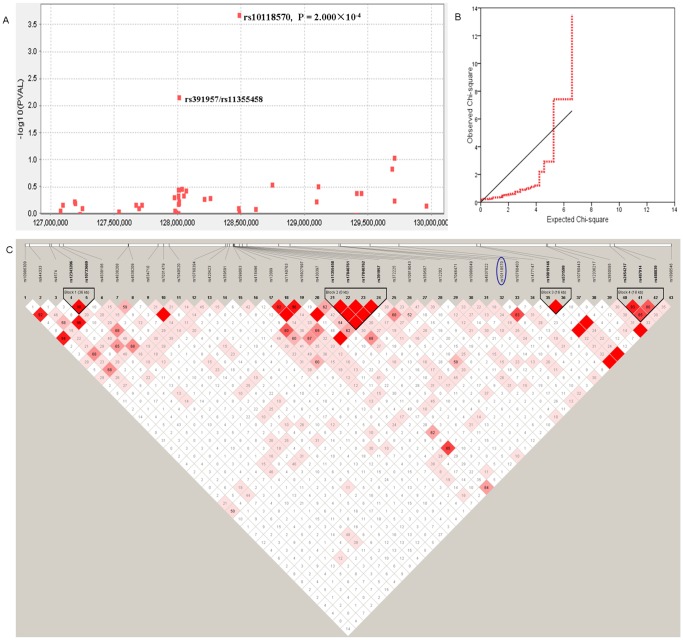
Case-control association and linkage disequilibrium (LD) on chromosome 9q33.3. (A) Manhattan plot displaying the results (−log_10_ of p-values) of the association of single-nucleotide polymorphisms (SNPs) with S. japonicum infection on 9q33.3 scan with respect to genomic position in the discovery study. The –log_10_ p values were for the association of each SNP with S. japonicum infection, from two-sided Cochran–Armitage tests for trend. (B) Quantile–quantile (Q–Q) plot for the test statistics of observed Chi-square values against expected Chi-square values were used to examine p value distributions based on the 43 SNPs in a hypothetical chromosome association study in the discovery cohort. (C) LD mapping on the chromosome 9q33.3 locus for the region including the 13 candidate genes (chromosome 9: 127,000,000–130,000,000) in the discovery study was visualized using default settings in Haploview version 4.2. Four blocks are designed according to the internally developed solid spine of LD. Block 1: rs12343206–rs10733669; Block 2: rs11355458–rs17840761–rs17840762–rs391957; Block 3: rs10819146–rs531599; Block 4: rs2454217–rs487914–rs488039. The value within each diamond represents the pairwise correlation between pairs of SNPs (measured as 100× D’) defined by the upper left and the upper right sides of the diamond. The diamond without a number corresponds to D’ = 1. Shading represents the magnitude and significance of D’, with a red-to-white gradient reflecting higher to lower D’ values. The black triangles indicate the location of haplotype blocks. The blue ellipse indicates the potential anti-infective SNP which not belongs to any block.

Two SNPs were excluded from further analysis due to unsuccessful genotyping. All SNPs conformed to Hardy-Weinberg equilibrium (data not shown). In the discovery study, we observed a suggestive association of SNPs rs10118570 (*P* = 2.000×10^−4^ in Figure 1A, and adjusted *P* = 2.807×10^−4^ in Table S1 in File S1), which located within *mitogen-activated protein kinase associated protein 1* (*MAPKAP1*). rs10118570 remained marginal significant after 10^5^ permutation tests (*P*_empirical_ = 0.0122; Table S1 in File S1). The distribution of *P*-values for each SNP was also compared to the expected distribution in a Q-Q plot where some deviation from expectation was observed at higher values (Figure 1B). The sharp deviation above an expected *X* ^2^ value of approximately 4.5 could be due to a strong association of the disease with SNPs on 9q33.3.

### Population attributable risk (PAR) in the discovery study

**Figure 2:**
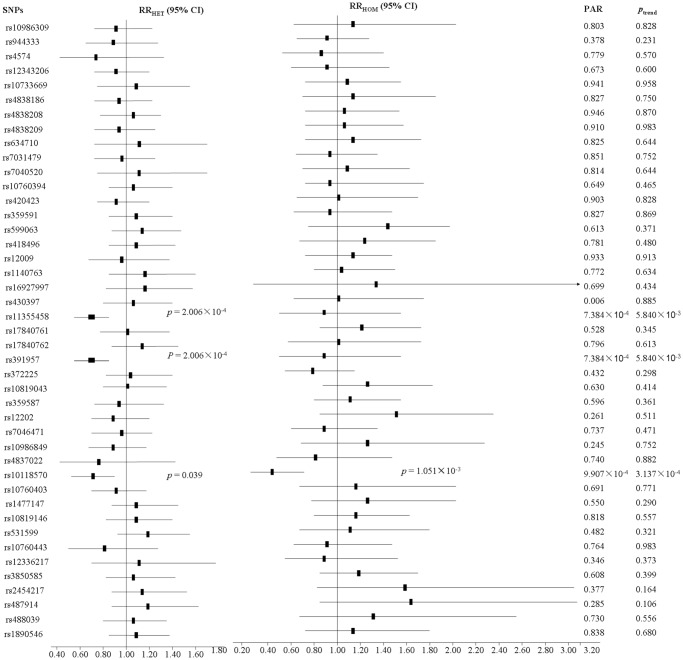
Results of single marker association analysis for the SNPs on 9q33.3 locus with S. japonicum infected risk in the discovery study. PARs (population attributable risks) were calculated in logistical regression models with adjustment for age, gender, smoking and drinking status. P_trend_ values were from the Cochran-Armitage trend test. PAR and P_trend_<1.163×10^−3^ means significant value by Bonferroni correction based on the total number of markers genotyped.

Although the highest PAR was rs391957 and rs11355458 (PAR = 7.384×10^−4^), their trends did not achieve significance (*P*_trend(s)_ = 5.840×10^−3^) by Bonferroni correction. We found both observed and predicted heterozygosities of rs10118570 in cases are lower than that in controls with a higher risk (PAR = 9.907×10^−4^) and a conspicuous trend (*P*_trend_ = 3.137×10^−4^). Furthermore, the homozygote GG had a low relative risk (RR_HOM_ = 0.42, 95% CI 0.24–0.73, *P* = 1.051×10^−3^; Figure 2).

### The haplotypes in 9q33.3 and *S. japonicum* infectious risk in the discovery study

To construct the haplotypes, we estimated the underlying haplotypic blocks of the 43 SNPs in both controls and cases. We identified four inheritance blocks. The block 1 is 36 kb and encompasses the promote of *PSMB7* to the seventh intron of *GPR144*; block 2 is within *GRP78*, including its 5′ UTR and promoter, and is only 236 bp; block 3 extends near 16.5 kb from FAM125B promoter to its first intron; while block 4 contains about 18.7 kb of intergenic sequence of the RALGPS1 (Figure 1C). The association analysis of the block data showed none of the haplotype was associated with infection of *S. japonicum.* But the diplotype Gd-CT-CC-AG in the block 2 achieved marginal level and showed slightly protective association (*P* = 0.0017; Table S2 in File S1).

### 9q33.3 and *S. japonicum* infectious risk with replication study

Because the LD analysis also revealed that rs391957 and rs11355458 were completely linked with pairwise D’ = 1 and r^2^ = 1 not only in controls but in cases (Figure 1C), we only chose the aforementioned two SNPs (rs391957 and rs10118570) for replicative and combined studies in the independent cohorts. Replicative and combined studies showed consistent association for rs10118570 genotype GG (replicative: adjusted OR 0.37, 95% CI 0.20–0.69, *P* = 9.581×10^−4^; Combined: adjusted OR 0.45, 95% CI 0.29–0.66, *P* = 7.902×10^−6^), while rs391957 did not achieve the significance level either in replicative cohort or in combined cohort (Table 3).

**Table 3:** Association results of the two SNPs with risk of *S. japonicum* infection in the discovery, replicative and combined studies.

SNPs	Discovery study				Replicative study				Combined study			
	Cases	Controls	OR	*P*	Cases	Controls	OR	*P*	Cases	Controls	OR	*P*
	(%)	(%)	(95% CI)		(%)	(%)	(95% CI)		(%)	(%)	(95% CI)	
rs391957												
AA	30	27	1.13	0.508	24	71	0.83	0.559	54	98	0.97	0.837
	(5.71)	(5.08)	(0.66–1.94)		(4.91)	(5.69)	(0.52–1.38)		(5.33)	(5.51)	(0.69–1.36)	
AG	167	230	0.69	0.003	210	490	1.12	0.187	377	720	0.87	0.087
	(31.81)	(43.23)	(0.51–0.86)		(42.94)	(39.29)	(0.92–1.43)		(37.18)	(40.47)	(0.74–1.02)	
GG	328	275	1.59	0.001	255	686	0.93	0.303	583	961	1.15	0.076
	(62.48)	(51.69)	(1.25–2.03)		(52.15)	(55.01)	(0.75–1.14)		(57.50)	(54.02)	(0.99–1.35)	
rs10118570												
GG	15	36	0.35	9.616×10^−4^	14	87	0.37	9.581×10^−4^	29	123	0.45	7.902×10^−6^
	(2.86)	(6.77)	(0.18–0.69)		(2.86)	(6.98)	(0.20–0.69)		(2.86)	(6.91)	(0.29–0.66)	
AG	150	179	0.82	0.086	206	449	1.27	0.039	356	628	0.93	0.808
	(28.57)	(33.65)	(0.62–1.05)		(42.13)	(36.01)	(1.04–1.55)		(35.11)	(35.30)	(0.80–1.13)	
AA	360	317	1.36	3.553×10^−3^	269	711	0.97	0.496	629	1028	1.21	0.025
	(68.57)	(59.59)	(1.10–1.84)		(55.01)	(57.02)	(0.77–1.18)		(62.03)	(57.79)	(1.03–1.44)	

Adjusted for age, gender, smoking and drinking.

*P*<1.163×10^−3^ means significant value by Bonferroni correction based on the total number of markers genotyped.

### 9q33.3 and risk of hepatic fibrosis (HF) and replication study

**Figure 3:**
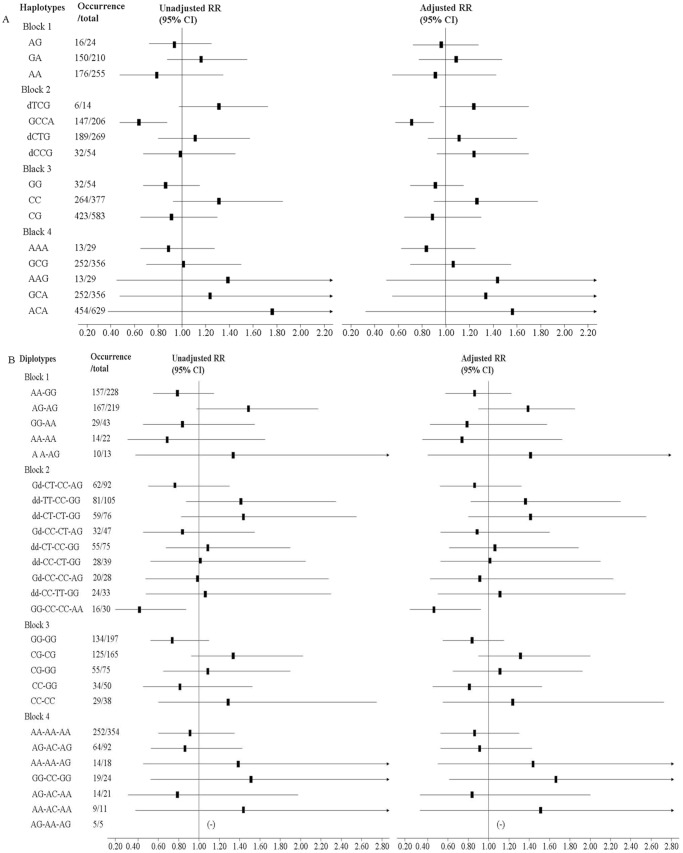
Association of (A) haplotypes and (B) diplotypes on 9q33.3 with risk of hepatic fibrosis in chronic S. japonicum infected Adults in the discovery study. Block 1, rs12343206–rs10733669; Block 2, rs11355458–rs17840761–rs17840762–rs391957; Block 3, rs10819146–rs531599; Block 4, rs2454217–rs487914–rs488039. RR, relative risk; CI, confidence interval. Unadjusted RR was calculated using Pearson Chi-square test. Adjusted RR was adjusted for age, gender, smoking, drinking and HBsAg. d, the deleted base.

The fibrosis was detected by hepatic imaging in 377 of 525 discovery cases (71.81%), 342 of 489 replicative cases (69.94%), and 719 of 1014 combined cases (70.91%) (Table 1). Risk analysis was performed with regard to fibrosis in subsets of cases with different genotypes, alleles, haplotypes and diplotypes. We could define both tentative protective roles [rs11355458 genotype GG and rs391957 genotype AA (Table S3 in File S1), block 2 haplotype GCCA and diplotype GG-CC-CC-AA (Figure 3)], and putative predisposing roles [rs11355458 deleted genotype and rs391957 genotype GG (Table S3 in File S1)] in the *GRP78* gene. Additionally, the results also showed that rs10118570 genotype GG was associated with anti-fibrogenesis (adjusted RR 0.44, 95% CI 0.20–0.93) (Table S3 in File S1).

**Figure 4:**
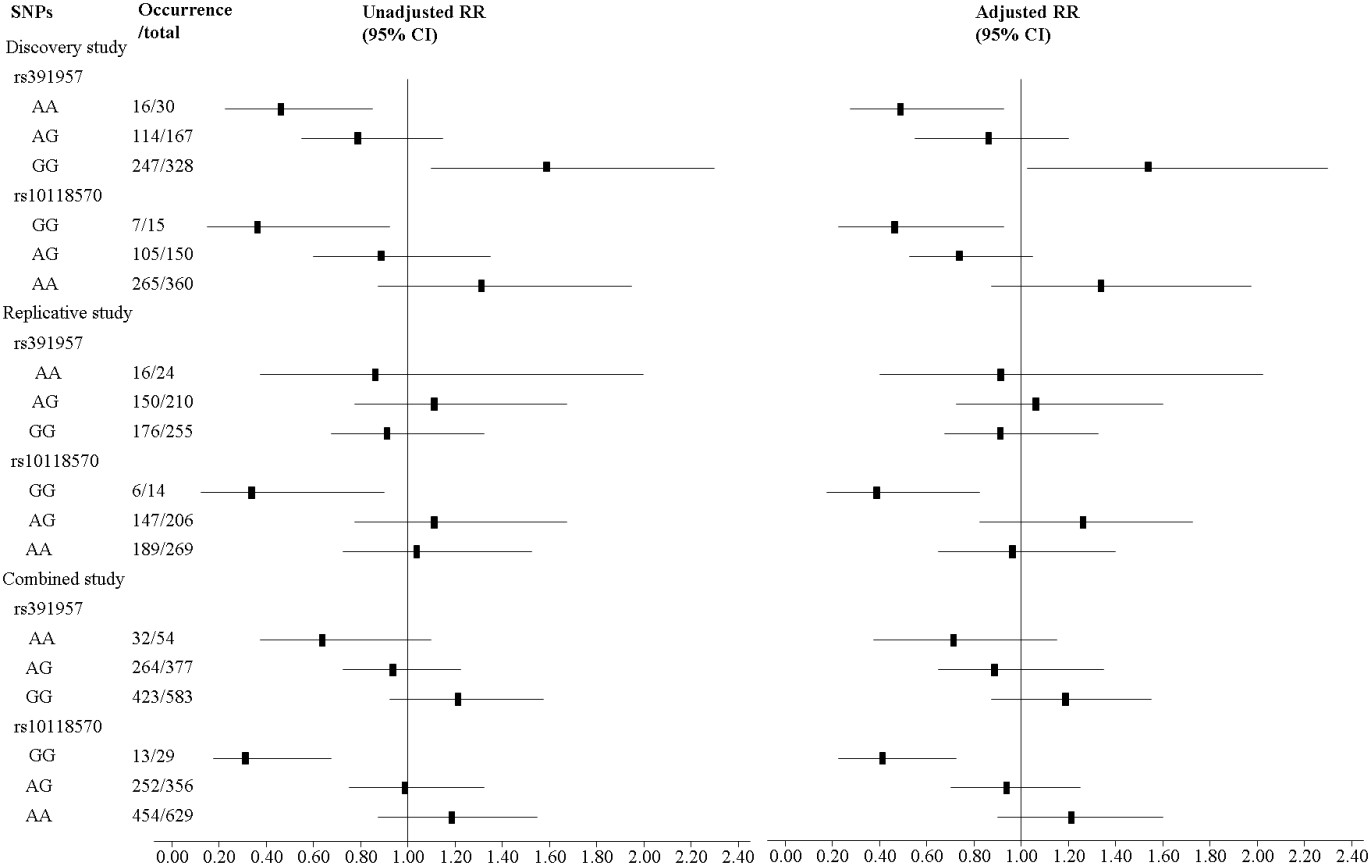
Association results of two SNPs with risk of hepatic fibrosis in chronic S. japonicum infected adults in the discovery, replication and combined studies. Unadjusted RR was calculated using Pearson Chi-square test. Adjusted RR was adjusted for age, gender, smoking, drinking and HBsAg. d, the deleted base.

Likewise, rs10118570 genotype GG had a strong protective quality both in the replicative cohort (adjusted RR 0.40, 95% CI 0.19–0.83) and in the combined cohort (adjusted RR 0.42, 95% CI 0.23–0.73); while rs391957 still did not achieve the significance level either in the replicative cohort or in the combined cohort (Figure 4).

### Independent prognostic factors analysis and replication study in HF patients

**Figure 5:**
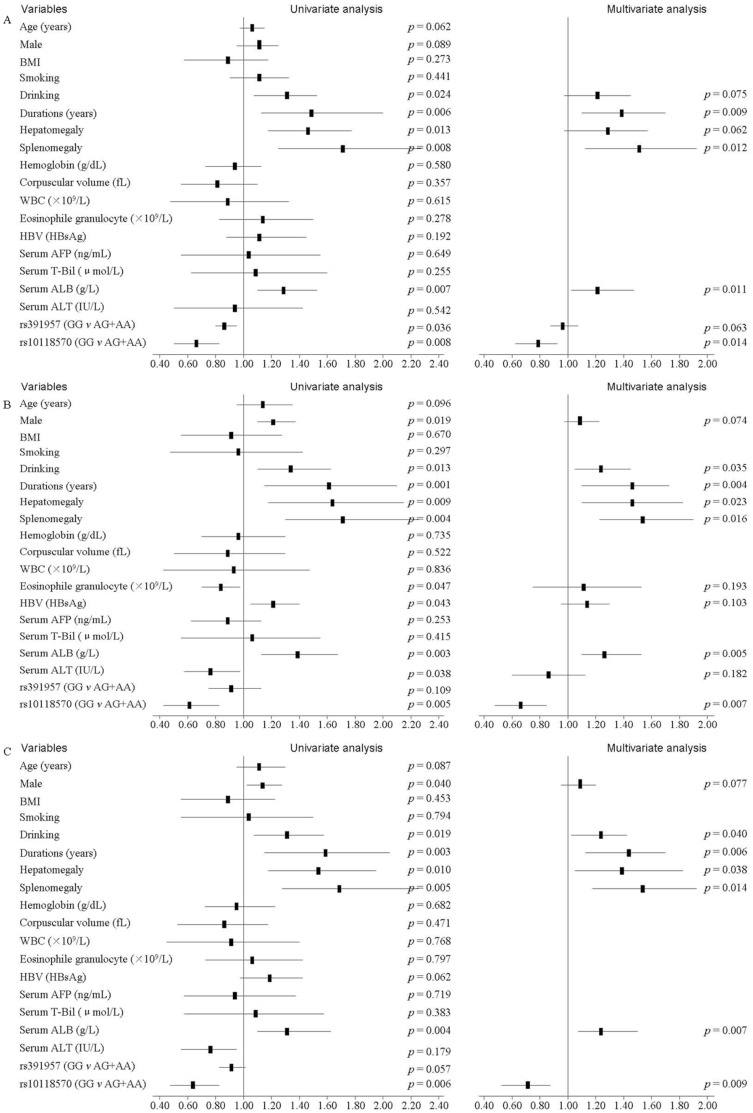
Variables associated with presence or absence of advanced hepatic fibrosis (grade III/IV) in the discovery, replicative and combined cohorts with univariate and multivariate analysis. (A) Analysis in the discovery cohort. (B) Analysis in the replicative cohort. (C) Analysis in the combined cohort.

In the discovery cohort, 148 (28.19%) patients did not have fibrosis, 287 (54.67%) had mild fibrosis (grade I or II), and 90 (17.14%) had advanced fibrosis (grade III or IV). In the replicative cohort, 147 (30.06%) patients did not have fibrosis, 256 (52.35%) had mild fibrosis, and 86 (17.59%) were advanced fibrosis. Of the total 1014 patients, 295 (29.09%) did not have fibrosis, 543 (53.55%) had mild fibrosis with I or II, and 176 (17.36%) had advanced fibrosis (Table 1). The univariate logistical and multivarite Cox regression models adjusted for other factors indicate that the duration time (years), splenomegaly, serum ALB and rs10118570 were recorded to be significantly different between advanced fibrosis group and mild fibrosis group (all *P*<0.05) in the discovery cohort (Figure 5A) and the replicative cohort (Figure 5B). Finally, we conducted a combined analysis of the two cohorts on advanced fibrosis. As shown in Figure 5C, the duration time (years), splenomegaly, serum ALB and rs10118570 remained significant independent predictors of advanced fibrosis using univarite and multivariate analysis. The HR for the duration time, splenomegaly, serum ALB and rs10118570 were 1.44 (*P* = 0.006), 1.54 (*P* = 0.014), 1.25 (*P* = 0.007) and 0.71 (*P* = 0.009), respectively.

### The interactions between rs10118570 and rs391957

Lastly, we investigated the statistical interactions between *MAPKAP1* (rs10118570) and *GRP78* (rs391957) with respect to schistosomiasis japonica in the discovery, replicative and combined cohorts. We found there was no direct interaction between the two SNPs in the replicative and combined cohort (all *P*_int_>0.0167) (Table 4). In addition, rs391957 and rs10118570 were not in LD either in controls or in cases in the three cohorts (Figure S1 in File S1). These results, thereby, strongly suggested that rs10118570 GG is an independent protective factor both in anti-infection and in anti-fibrosis.

**Table 4:** Analysis of the statistical interactions between rs10118570 and rs391957 in the discovery, replicative and combined cohorts.

	rs391957 AA		OR 95% CI	*P*_int_ *	rs391957 AG		OR 95% CI	*P*_int_ *	rs391957 GG		OR 95% CI	*P*_int_ *
rs10118570	Cases	Controls			Cases	Controls			Cases	Controls		
Discovery												
GG	1	5	0.15 (0.02–1.39)	0.062	0	12	/	/	14	19	0.60 (0.30–1.22)	0.156
AG	6	3	2.00 (0.45–8.94)	0.358	50	70	0.98 (0.63–1.51)	0.916	104	106	0.74 (0.53–1.04)	0.079
AA	23	19	1.38 (0.42–4.51)	0.59	117	148	1.30 (0.85–1.99)	0.233	210	150	1.48 (1.07–2.06)	0.018
Replicative												
GG	1	8	0.34 (0.04–2.89)	0.304	0	38	/	/	13	41	0.85 (0.45–1.60)	0.606
AG	8	17	1.59 (0.58–4.36)	0.366	82	159	1.33 (0.95–1.87)	0.092	116	273	1.26 (0.95–1.69)	0.115
AA	15	46	0.91 (0.35–2.36)	0.84	128	293	1.05 (0.75–1.46)	0.775	126	372	0.82 (0.62–1.10)	0.188
Combined												
GG	2	13	0.25 (0.06–1.16)	0.059	0	50	/	/	27	60	0.73 (0.46–1.16)	0.183
AG	14	20	1.37 (0.62–2.98)	0.435	132	229	1.16 (0.89–1.50)	0.283	220	379	0.93 (0.75–1.15)	0.506
AA	38	65	1.21 (0.59–2.47)	0.61	245	441	1.17 (0.91–1.52)	0.225	336	532	1.14 (0.93–1.41)	0.204

*P_int_, *P* value for the interaction.

*P*_int_<0.0167 means significant value by Bonferroni correction based on the total number of studies.

## Discussion

This study addressed the molecular epidemiology of chronic schistosomiasis in people living in communities in China. We analyzed 43 SNPs in total 1014 cases and 1779 controls from two independent cohorts. The studies were located at two different areas within known endemic regions of the country. The two areas located in the watersheds of the Yangtz River and its tributary were beyond 500 km of one another. In the cohorts, age and gender were important demographic factors of HF. The Males have markedly higher prevalence of HF compared with that in females, which was expected as previous studies [30], [31]. Patients with HF have poor nutritional status with lower albumin in serum. Hypoalbuminemia is perhaps imputed to reducing hepatic synthesis and intestinal malabsorption [32]. In addition, liver function is almost normal or a little abnormal during chronic schistosomiasis because of standard treatment with Praziquantel in China.

The studies of schistosomiasis have focused on HF and hepatosplenic disease. Periportal fibrosis of the liver is the most severe symptom in chronic schistosomiasis patients, eventually resulting in portal hypertension with subsequent hepatosplenomegaly and esophageal varices [33], [34], [35]. Infection by *S. japonicum* could induce oxidative stress, which is an imbalance between reactive oxygen species (ROS) production and the ability of organism to promptly detoxicate these reactive intermediates or facilely repair the resulting damage. In fact, ROS can cause DNA, protein and lipid damage, and HF often show increased levels of DNA base oxidation and mutations [36]. Thus, oxidative stress is received as a crucial mechanism in pathophysiological changes, including fibrosis formation in liver.

Since vaccines for schistosomiasis are not yet available, drugs remains the key method of controlling the damaging effects of the infection. The normalized chemotherapy could be extremely helpful in areas of high transmission where people could be infected even at a very young age. The earlier the chemotherapy, the slower the fibrogenesis is [37]. The patients in our studies were all chronic hospitalized patients and received regular treatment. Data from our studies have revealed that the nutritional status, such as body mass index (BMI), and other clinical index in non-HF cases were better than that in HF cases, accompanied by decrease in liver or spleen bulk and a reduction in the portal pressure. It is however, important to notice that the absence of ultrasonographic HF does not obviate the possible presence of severe morbidity in *S. japonicum* infected patients.

We identified a strong association of *MAPKAP1* (rs10118570) with anti-infection and anti-fibrosis in chronic schistosomiasis japonica. *MAPKAP1* polymorphisms were significantly associated with the prevalence of subarachnoid hemorrhage [38]. Nonetheless, its associations with other diseases were not reported. *MAPKAP1* encodes a protein that is highly similar to the yeast SIN1 protein [39]. As a stress-activated protein kinase, it could inhibit mitogen-activated protein kinase kinase kinase 2 (MAP3K2), platelet-derived growth factor receptor beta (PDGFRbeta) and K-ras signaling [40], [41], [42], and enhance osmotic stress-induced phosphorylation of activation transcription factor (ATF-2) and ATF2-mediated transcription [43].

Knowledge of the role of *MAPKAP1* in schistosomiasis triggers efforts to clarify the mechanisms of HF and hepatosplenic disease. Since fibrosis induced by *S. japonicum* infection presented over-expressions of PDGF [44], and also, the difference of activated p38 MAPK and ATF-2 may explain the function of cells from hepatosplenic and intestinal patients [45]. Therefore, we infer that MAPKAP1 would play a central role by receiving and responding to input stress signals from various pathways and regulating transduction of signals in the hepatic fibrosis pathogenesis.

In summary, we have identified a novel association between *MAPKAP1* rs10118570 and chronic infection with *S. japonicum* and fibrosis. And *MAPKAP1* may represent a novel anti-fibrosis gene influencing fibrosis in chronic schistosomiasis japonica including other stress-related syndromes. The causal mechanisms are not yet known, but the 9q33.3 locus has pleiotropic effects on physiological and pathological stress. This is robust evidence that the widely described association between 9q33.3 and stress-associated diseases has genetic components. The approach searching consistent effects on all three cohorts used in this study may represent an effective method to discover association of pleiotropy, although, further investigation of the mechanism of *MAPKAP1* regulation is still warranted. That will illuminate the biological pathways important for anti-infection and anti-fibrosis in chronic Schistosomiasis japonica.

## Supporting Information

File S1 Figure S1 - Linkage disequilibrium (LD) mapping of rs391957 and rs10118570 showed that these two SNPs were NOT linked. Table S1 - Association of candidate gene SNPs on chromosome 9q33.3 with S. japonicum in the discovery study. Table S2 - Association of haplotypes and diplotypes on 9q33.3 with risk of S. japonicum infection in the discovery study. Table S3 - Association of the genotypes on 9q33.3 with risk of hepatic fibrosis in chronic S. japonicum infected adults in the discovery study.
(DOC)
