# Supplementary material for: MAPKAP1 rs10118570 Polymorphism Is Associated with Anti-Infection and Anti-Hepatic Fibrogenesis in Schistosomiasis Japonica
Source: PLoS One. 2014 Aug 25;9(8):e105995. doi: 10.1371/journal.pone.0105995 (PMC4143368; doi:10.1371/journal.pone.0105995)
Supplement: File S1 — Figure S1 - Linkage disequilibrium (LD) mapping of rs391957 and rs10118570 showed that these two SNPs were NOT linked. Table S1 - Association of candidate gene SNPs on chromosome 9q33.3 with S. japonicum in the discovery study. Table S2 - Association of haplotypes and diplotypes on 9q33.3 with risk of S. japonicum infection in the discovery study. Table S3 - Association of the genotypes on 9q33.3 with risk of hepatic fibrosis in chronic S. japonicum infected adults in the discovery study. (DOC) [file pone.0105995.s001.doc]

***MAPKAP1* rs10118570 Polymorphism is Associated with Anti-Infection and Anti-Hepatic Fibrogenesis in Schistosomiasis Japonica**

Xiao Zhu^1,2a^*, Jinfang Zhang^3a^, Wenguo Fan^4a^, Yunguo Gong^5^, Jianhua Yan^6^, Zhidong Yuan^7^, Lang Wu^8^, Hongjing Cui^1^, Haiqing Luo^1^, Qingming Kong^9^, Li Tang^10^, Shuilong Leng^3,11^, Yufeng Liao^12^, Weiming Fu^3,13^, Qin Xiao^2^*, Dongpei Li^1,14^*

**1** Guangdong Province Key Laboratory of Medical Molecular Diagnosis, Department of Clinical Oncology, Guangdong Medical College, Zhanjiang/Dongguan, China, **2** Peking University Shenzhen Hospital, Shenzhen Peking University - The Hong Kong University of Science and Technology Medical Center, Shenzhen, China, **3** Department of Orthopaedics & Traumatology, The Chinese University of Hong Kong, Prince of Wales Hospital, Hong Kong, China, **4** Guangdong Provincial Key Laboratory of Stomatology, Guanghua School of Stomatology, Sun Yat-sen University, Guangzhou, China, **5** School of Biomedical Informatics, University of Texas, Houston, TX 77030, USA, **6** Clinical Imaging Research Center, A*STAR-NUS, Singapore 117599, **7**School of Life Sciences, Hunan University of Science and Technology, Xiangtan, China, **8**Center for Clinical and Translational Science, Mayo Clinic, Rochester, MN 55905, USA, **9** Immunity and Biochemical Research Lab, Zhejiang Academy of Medical Sciences, Hangzhou, China, **10** Schistosomiasis Institute, Hubei Academy of Preventive Medicine, Hubei Provincial Center for Disease Control and Prevention, Wuhan, China, **11** Department of Human Anatomy, Guangzhou Medical University, Guangzhou, China, **12** Department of Laboratory Medicine, Ningbo No.2 Hospital, Ningbo, China, **13** Guangzhou Institute of Advanced Technology, Chinese Academy of Sciences, Guangzhou, China, **14** Institute of Molecular Medicine and Genetics, Department of Neuroscience and Regenerative Medicine, Georgia Regents University, Augusta, GA 30912, USA

**Supporting Information**

Table S1

Table S2

Table S3

Figure S1

**Table S1.** Association of candidate gene SNPs on chromosome 9q33.3 with S. japonicum in the discovery study.

| SNPs | Chr.  location^*^ | Genes | Types | A>B^†^ | C^‡^ | Allele ratios:  Cases, Controls^§^ | Frequency:  Cases, Control|| | *p*^¶^ | Permutation Analysis^#^ | |
| --- | --- | --- | --- | --- | --- | --- | --- | --- | --- | --- |
|  |  |  |  |  |  |  |  |  | *X^2^* | *p*_empirical_ |
| rs10986309 | 127067131 | NEK6 | Intron | G>A | A | 243:807, 242:822 | 0.231, 0.227 | 0.786 | 0.047 | 1 |
| rs944333 | 127087002 | NEK6 | Intron | A>G | G | 449:601, 444:620 | 0.428, 0.417 | 0.519 | 0.231 | 1 |
| rs4574 | 127177161 | PSMB7 | V39A | T>C | C | 243:807, 235:829 | 0.231, 0.221 | 0.284 | 0.337 | 1 |
| rs12343206 | 127184067 | PSMB7 | Promoter | A>G | A | 745:305, 744:320 | 0.710, 0.699 | 0.883 | 0.268 | 1 |
| rs10733669 | 127220133 | GPR144 | Intron | G>A | A | 688:362, 696:368 | 0.655, 0.654 | 0.717 | 0.003 | 1 |
| rs4838186 | 127238332 | GPR144 | Intron | C>T | T | 267:783, 264:800 | 0.254, 0.248 | 0.643 | 0.107 | 1 |
| rs4838208 | 127528035 | NR6A1 | Intron | A>C | C | 355:695, 356:708 | 0.338, 0.335 | 0.253 | 0.029 | 1 |
| rs4838209 | 127528842 | NR6A1 | Intron | T>C | T | 711:339, 720:344 | 0.677, 0.677 | 0.959 | 0.001 | 1 |
| rs634710 | 127661645 | GOLGA1 | T425M | T>C | T | 756:294, 756:308 | 0.720, 0.711 | 0.510 | 0.233 | 1 |
| rs7031479 | 127686126 | GOLGA1 | Intron | T>C | T | 698:352, 700:364 | 0.665, 0.658 | 0.401 | 0.111 | 1 |
| rs7040520 | 127711321 | GOLGA1 | Promoter | A>G | A | 756:294, 756:308 | 0.720, 0.711 | 0.815 | 0.233 | 1 |
| rs10760394 | 127962533 | RABEPK | Promoter | G>A | A | 329:721, 318:746 | 0.313, 0.299 | 0.686 | 0.520 | 1 |
| rs420423 | 127969785 | RABEPK | Intron (boundary) | T>C | T | 777:273, 783:281 | 0.740, 0.736 | 0.432 | 0.046 | 1 |
| rs359591 | 127979491 | RABEPK | Intron | A>G | G | 341:709, 342:722 | 0.325, 0.321 | 0.654 | 0.027 | 1 |
| rs599063 | 127996046 | GRP78 | Downstream | A>G | G | 237:813, 222:842 | 0.226, 0.209 | 0.310 | 0.906 | 1 |
| rs418496 | 127996479 | GRP78 | Downstream | G>A | A | 218:832, 207:857 | 0.208, 0.195 | 0.296 | 0.562 | 1 |
| rs12009 | 127997303 | GRP78 | 3' UTR | T>C | T | 646:404, 652:412 | 0.615, 0.613 | 0.847 | 0.013 | 1 |
| rs1140763 | 127997592 | GRP78 | 3' UTR | T>C | T | 570:480, 566:498 | 0.543, 0.532 | 0.512 | 0.253 | 1 |
| rs16927997 | 127998397 | GRP78 | 3' UTR | T>C | C | 102:948, 93:971 | 0.097, 0.087 | 0.673 | 0.598 | 1 |
| rs430397 | 128001119 | GRP78 | Intron (boundary) | G>A | A | 220:830, 212:852 | 0.210, 0.199 | 0.305 | 0.343 | 1 |
| rs11355458 | 128003789 | GRP78 | 5' UTR | ^ζ^d>G | G | 823:227, 780:284 | 0.784, 0.733 | 0.009 | 7.419 | 0.2359 |
| rs17840761 | 128003979 | GRP78 | Promoter | C>T | T | 452:598, 435:629 | 0.430, 0.409 | 0.416 | 1.016 | 1 |
| rs17840762 | 128003987 | GRP78 | Promoter | C>T | T | 227:823, 219:845 | 0.216, 0.206 | 0.264 | 0.341 | 1 |
| rs391957 | 128004024 | GRP78 | Promoter | G>A | A | 823:227, 780:284 | 0.784, 0.733 | 0.009 | 7.419 | 0.2359 |
| rs372225 | 128024049 | GAPVD1 | Promoter | C>T | C | 577:473, 562:502 | 0.550, 0.528 | 0.293 | 0.967 | 1 |
| rs10819043 | 128037245 | GAPVD1 | Intron | T>C | C | 419:631, 407:657 | 0.399, 0.383 | 0.330 | 0.606 | 1 |
| rs359587 | 128058377 | GAPVD1 | Intron | T>A | T | 586:464, 572:492 | 0.558, 0.538 | 0.391 | 0.897 | 1 |
| rs12202 | 128200299 | MAPKAP1 | 3' UTR | G>C | C | 240:810, 230:834 | 0.229, 0.216 | 0.505 | 0.470 | 1 |
| rs7046471 | 128250409 | MAPKAP1 | Intron | T>C | T | 710:340, 704:360 | 0.676, 0.662 | 0.552 | 0.504 | 1 |
| rs10986849 | 128469683 | MAPKAP1 | Promoter | A>G | A | 829:221, 834:230 | 0.790, 0.784 | 0.614 | 0.102 | 1 |
| rs4837022 | 128475379 | MAPKAP1 | Promoter | C>T | T | 211:839, 211:853 | 0.201, 0.198 | 0.708 | 0.023 | 1 |
| rs10118570 | 128476464 | MAPKAP1 | Promoter | A>G | G | 870:180, 813:251 | 0.829, 0.764 | 2.807×10^-4^ | 13.534 | 0.0122 |
| rs10760403 | 128608495 | PBX3 | Intron | T>G | T | 862:188, 868:196 | 0.821, 0.816 | 0.623 | 0.095 | 1 |
| rs1477147 | 128736511 | PBX3 | Downstream | C>G | G | 250:800, 232:832 | 0.238, 0.218 | 0.255 | 1.207 | 0.9999 |
| rs10819146 | 129084932 | FAM125B | Promoter | G>C | C | 416:634, 408:656 | 0.396, 0.383 | 0.501 | 0.360 | 1 |
| rs531599 | 129101389 | FAM125B | Intron | G>C | C | 241:809, 224:840 | 0.230, 0.211 | 0.298 | 1.112 | 1 |
| rs10760443 | 129395346 | LMX1B | Intron | T>C | C | 332:718, 336:728 | 0.316, 0.316 | 0.845 | 0.001 | 1 |
| rs12336217 | 129399870 | LMX1B | Intron | A>G | G | 333:717, 319:745 | 0.317, 0.300 | 0.590 | 0.744 | 1 |
| rs3850585 | 129436272 | LMX1B | Intron | C>T | T | 414:636, 400:664 | 0.394, 0.376 | 0.343 | 0.751 | 1 |
| rs2454217 | 129679933 | RALGPS1 | Intron | A>G | G | 166:884, 144:920 | 0.158, 0.135 | 0.172 | 2.187 | 0.9927 |
| rs487914 | 129698639 | RALGPS1 | Intron | A>C | C | 172:878, 146:918 | 0.164, 0.137 | 0.096 | 2.924 | 0.9541 |
| rs488039 | 129698676 | RALGPS1 | Intron | A>G | G | 159:891, 151:913 | 0.151, 0.142 | 0.594 | 0.382 | 1 |
| rs1890546 | 129952141 | RALGPS1 | Intron | T>C | C | 264:786, 259:805 | 0.251, 0.243 | 0.583 | 0.182 | 1 |

^*^Positions according to genomic contig NT_008470.18 (Entrez Nucleotide) in National Center for Biotechnology Information Genome Build 36.2.

^†^The major allele is listed first, then the minor allele. ^‡^Potential causal allele.

^§^Number of alleles were compared in cases versus controls: allele(1):allele(2) cases, allele(1):allele(2) controls.

^||^Frequency of the association allele.

^¶^Calculated in logistical regression models with adjustment for age, gender, smoking and drinking status; p < 1.163×10^-3^ means significant value by Bonferroni correction based on the total number of markers genotyped.

^#^Empirical *P*-value based on 10^5^ permutations of case-control status using the max(T) procedure. p < 0.05 means significant value.

^ζ^d, the delete allele.

**Table S2.** Association of haplotypes and diplotypes on 9q33.3 with risk of S. japonicum infection in the discovery study.

|  | Cases (%) | Controls (%) | OR (95% CI)^†^ | *P*^†^ |
| --- | --- | --- | --- | --- |
| Haplotypes^‡^ |  |  |  |  |
| Block 1 |  |  |  |  |
| AG | 688 (65.52) | 695 (65.32) | 1.03 (0.85-1.24) | 0.893 |
| GA | 305 (29.05) | 316 (29.70) | 0.94 (0.77-1.15) | 0.707 |
| AA | 57 (5.43) | 53 (4.98) | 1.09 (0.75-1.60) | 0.659 |
| *P*_trend_|| |  |  | 0.947 |  |
| Block 2^¶^ |  |  |  |  |
| dTCG | 452 (43.05) | 440 (41.35) | 1.10 (0.91-1.30) | 0.385 |
| GCCA | 227 (21.62) | 279 (26.22) | 0.79 (0.65-0.97) | 0.019 |
| dCTG | 227 (21.62) | 214 (20.11) | 1.10 (0.88-1.35) | 0.37 |
| dCCG | 144 (13.71) | 131 (12.31) | 1.10 (0.86-1.42) | 0.358 |
| *P*_trend_|| |  |  | 0.575 |  |
| Block 3 |  |  |  |  |
| GG | 634 (60.38) | 656 (61.65) | 0.94 (0.78-1.12) | 0.522 |
| CC | 241 (22.95) | 224 (21.05) | 1.13 (0.91-1.38) | 0.247 |
| CG | 175 (16.67) | 184 (17.29) | 0.93 (0.74-1.17) | 0.656 |
| *P*_trend_|| |  |  | 0.846 |  |
| Block 4 |  |  |  |  |
| AAA | 855 (81.43) | 887 (83.36) | 0.87 (0.69-1.10) | 0.219 |
| GCG | 136 (12.95) | 133 (12.50) | 1.07 (0.82-1.38) | 0.684 |
| AAG | 18 (1.71) | 20 (1.88) | 0.93 (0.50-1.76) | 0.833 |
| GCA | 25 (2.38) | 10 (0.94) | 2.49 (1.19-5.35) | 0.01 |
| ACA | 11 (1.05) | 13 (1.22) | 0.86 (0.38-1.93) | 0.691 |
| *P*_trend_|| |  |  | 0.194 |  |
| Diplotypes^§^ |  |  |  |  |
| Block 1 |  |  |  |  |
| AA-GG | 228 (43.43) | 232 (43.61) | 0.99 (0.78-1.27) | 0.891 |
| AG-AG | 219 (41.71) | 223 (41.92) | 0.99 (0.78-1.27) | 0.917 |
| GG-AA | 43 (8.19) | 47 (8.83) | 0.92 (0.60-1.42) | 0.723 |
| AA-AA | 22 (4.19) | 20 (3.76) | 1.12 (0.60-2.08) | 0.494 |
| A A-AG | 13 (2.48) | 10 (1.88) | 1.33 (0.58-3.05) | 0.386 |
| *P*_trend_|| |  |  | 0.697 |  |
| Block 2^¶^ |  |  |  |  |
| Gd-CT-CC-AG | 92 (17.52) | 136 (25.56) | 0.62 (0.46-0.83) | 0.0017 |
| dd-TT-CC-GG | 105 (20.00) | 93 (17.48) | 1.18 (0.87-1.61) | 0.365 |
| dd-CT-CT-GG | 76 (14.48) | 62 (11.65) | 1.28 (0.90-1.84) | 0.124 |
| Gd-CC-CT-AG | 47 (8.95) | 60 (11.28) | 0.77 (0.52-1.16) | 0.192 |
| dd-CT-CC-GG | 75 (14.29) | 53 (9.96) | 1.51 (1.04-2.19) | 0.085 |
| dd-CC-CT-GG | 39 (7.43) | 35 (6.58) | 1.14 (0.71-1.83) | 0.494 |
| Gd-CC-CC-AG | 28 (5.33) | 34 (6.39) | 0.83 (0.49-1.38) | 0.61 |
| dd-CC-TT-GG | 33 (6.29) | 32 (6.02) | 1.05 (0.63-1.73) | 0.817 |
| GG-CC-CC-AA | 30 (5.71) | 27 (5.08) | 1.13 (0.66-1.94) | 0.508 |
| *P*_trend_|| |  |  | 0.122 |  |
| Block 3 |  |  |  |  |
| GG-GG | 197 (37.52) | 208 (39.10) | 0.94 (0.73-1.20) | 0.647 |
| CG-CG | 165 (31.43) | 152 (28.57) | 1.15 (0.88-1.49) | 0.26 |
| CG-GG | 75 (14.29) | 88 (16.54) | 0.84 (0.60-1.18) | 0.267 |
| CC-GG | 50 (9.52) | 48 (9.02) | 1.06 (0.70-1.61) | 0.79 |
| CC-CC | 38 (7.24) | 36 (6.77) | 1.08 (0.67-1.73) | 0.803 |
| *P*_trend_|| |  |  | 0.819 |  |
| Block 4 |  |  |  |  |
| AA-AA-AA | 354 (67.43) | 371 (69.74) | 0.90 (0.69-1.17) | 0.378 |
| AG-AC-AG | 92 (17.52) | 90 (16.92) | 1.04 (0.76-1.44) | 0.854 |
| AA-AA-AG | 18 (3.43) | 20 (3.76) | 0.91 (0.48-1.74) | 0.599 |
| GG-CC-GG | 24 (4.57) | 16 (3.01) | 1.55 (0.81-2.94) | 0.165 |
| AG-AC-AA | 21 (4.00) | 14 (2.63) | 1.54 (0.78-3.07) | 0.386 |
| AA-AC-AA | 11 (2.10) | 13 (2.44) | 0.85 (0.38-1.93) | 0.706 |
| AG-AA-AG | 5 (0.95) | 8 (1.50) | 0.63 (0.21-1.94) | 0.323 |
| *P*_trend_|| |  |  | 0.533 |  |

*Block 1, rs12343206-rs10733669; Block 2, rs11355458-rs17840761-rs17840762-rs391957; Block 3, rs10819146-rs531599; Block 4, rs2454217-rs487914-rs488039.

†Calculated in logistical regression models with adjustment for age, gender, smoking and drinking status;

^‡^*P* < 0.0033 means significant value by Bonferroni correction based on the total number of markers of haplotypes.

^§^*P* < 0.0019 means significant value by Bonferroni correction based on the total number of markers of diplotypes.

^||^*P*_trend_ value from the Cochran-Armitage trend test.

^¶^d, the deleted allele.

**Table S3.** Association of the genotypes on 9q33.3 with risk of hepatic fibrosis in chronic S. japonicum infected adults in the discovery study.

| rs ID | Genotypes | HF/(HF+non-HF) (%) | RR (95% CI)^*,†^ | Adjusted RR (95% CI)^*,‡^ |
| --- | --- | --- | --- | --- |
| rs10986309 | AA | 22/30 (73.33) | 1.09 (0.47-2.49) | 1.04 (0.59-1.80) |
|  | AG | 129/183 (70.49) | 0.91 (0.61-1.35) | 0.81 (0.62-1.08) |
|  | GG | 226/312 (72.44) | 1.08 (0.73-1.59) | 1.25 (0.94-1.59) |
| rs944333 | AA | 126/190 (66.32) | 0.66 (0.45-0.97) | 0.79 (0.57-1.05) |
|  | AG | 165/221 (74.66) | 1.28 (0.87-1.89) | 1.14 (0.82-1.55) |
|  | GG | 86/114 (75.44) | 1.27 (0.79-2.04) | 1.17 (0.85-1.54) |
| rs4574 | TT | 236/316 (74.68) | 1.42 (0.97-2.09) | 1.32 (0.94-1.78) |
|  | CT | 118/175 (67.43) | 0.73 (0.49-1.08) | 0.80 (0.67-1.16) |
|  | CC | 23/34 (67.65) | 0.81 (0.38-1.71) | 0.91 (0.50-1.83) |
| rs12343206 | GG | 29/43 (67.44) | 0.80 (0.41-1.56) | 0.75 (0.39-1.55) |
|  | AG | 167/219 (76.26) | 1.47 (0.99-2.18) | 1.37 (0.93-2.05) |
|  | AA | 181/263 (68.82) | 0.74 (0.51-1.09) | 0.78 (0.53-1.13) |
| rs10733669 | GG | 157/228 (68.86) | 0.77 (0.53-1.13) | 0.82 (0.54-1.20) |
|  | AG | 177/232 (76.29) | 1.50 (1.01-2.21) | 1.39 (0.93-2.10) |
|  | AA | 43/65 (66.15) | 0.74 (0.42-1.28) | 0.83 (0.48-1.36) |
| rs4838186 | TT | 27/40 (67.50) | 0.80 (0.40-1.60) | 0.88 (0.56-1.54) |
|  | CT | 129/187 (68.98) | 0.81 (0.55-1.20) | 0.87 (0.58-1.22) |
|  | CC | 221/298 (74.16) | 1.31 (0.89-1.91) | 1.18 (0.91-1.53) |
| rs4838208 | CC | 49/70 (70.00) | 0.90 (0.52-1.57) | 0.88 (0.61-1.48) |
|  | AC | 157/215 (73.02) | 1.11 (0.75-1.63) | 1.15 (0.85-1.48) |
|  | AA | 171/240 (71.25) | 0.95 (0.65-1.39) | 0.91 (0.61-1.26) |
| rs4838209 | CC | 37/60 (61.67) | 0.59 (0.34-1.04) | 0.86 (0.60-1.21) |
|  | CT | 164/219 (74.89) | 1.30 (0.88-1.92) | 1.20 (0.93-1.54) |
|  | TT | 176/246 (71.54) | 0.98 (0.67-1.43) | 0.87 (0.68-1.12) |
| rs634710 | TT | 207/281 (73.67) | 1.22 (0.83-1.78) | 1.25 (0.90-1.65) |
|  | CT | 136/194 (70.10) | 0.88 (0.59-1.30) | 0.86 (0.68-1.17) |
|  | CC | 34/50 (68.00) | 0.82 (0.44-1.53) | 0.85 (0.58-1.30) |
| rs7031479 | CC | 56/73 (76.71) | 1.34 (0.75-2.40) | 1.17 (0.63-2.13) |
|  | CT | 151/206 (73.30) | 1.13 (0.76-1.67) | 1.11 (0.85-1.44) |
|  | TT | 170/246 (69.11) | 0.79 (0.53-1.14) | 0.88 (0.61-1.26) |
| rs7040520 | AA | 198/281 (70.46) | 0.87 (0.59-1.27) | 0.75 (0.57-1.19) |
|  | AG | 140/194 (72.16) | 1.03 (0.69-1.53) | 1.17 (0.79-1.51) |
|  | GG | 39/50 (78.00) | 1.44 (0.72-2.89) | 1.24 (0.80-2.12) |
| rs10760394 | AA | 28/47 (59.57) | 0.55 (0.29-1.01) | 0.70 (0.31-1.20) |
|  | AG | 166/235 (70.64) | 0.90 (0.62-1.32) | 0.91 (0.62-1.33) |
|  | GG | 183/243 (75.31) | 1.38 (0.94-2.03) | 1.33 (0.87-1.91) |
| rs420423 | CC | 25/33 (75.76) | 1.24 (0.55-2.82) | 1.15 (0.48-2.58) |
|  | CT | 139/207 (67.15) | 0.69 (0.47-1.01) | 0.78 (0.57-1.11) |
|  | TT | 213/285 (74.74) | 1.37 (0.94-2.01) | 1.29 (0.89-1.89) |
| rs359591 | GG | 34/52 (65.38) | 0.72 (0.39-1.31) | 0.92 (0.60-1.45) |
|  | AG | 167/237 (70.46) | 0.89 (0.61-1.30) | 0.86 (0.65-1.28) |
|  | AA | 176/236 (74.58) | 1.28 (0.87-1.89) | 1.19 (0.76-2.08) |
| rs599063 | GG | 26/39 (66.67) | 0.77 (0.38-1.54) | 0.70 (0.35-1.49) |
|  | AG | 110/159 (69.18) | 0.83 (0.55-1.25) | 0.80 (0.52-1.19) |
|  | AA | 241/327 (73.70) | 1.28 (0.87-1.88) | 1.32 (0.84-2.00) |
| rs418496 | AA | 28/33 (84.85) | 2.30 (0.87-6.06) | 2.15 (0.75-5.96) |
|  | AG | 114/152 (75.00) | 1.26 (0.82-1.93) | 1.33 (0.87-2.23) |
|  | GG | 235/340 (69.12) | 0.68 (0.45-1.02) | 0.76 (0.51-1.06) |
| rs12009 | TT | 151/216 (69.91) | 0.85 (0.58-1.25) | 0.93 (0.71-1.29) |
|  | CT | 164/214 (76.64) | 1.51 (1.02-2.24) | 1.43 (0.96-2.20) |
|  | CC | 62/95 (65.26) | 0.69 (0.43-1.10) | 0.65 (0.41-1.09) |
| rs1140763 | TT | 113/167 (67.66) | 0.75 (0.50-1.11) | 0.81 (0.62-1.07) |
|  | CT | 170/236 (72.03) | 1.02 (0.70-1.50) | 1.09 (0.83-1.43) |
|  | CC | 94/122 (77.05) | 1.42 (0.89-2.28) | 1.21 (0.75-2.04) |
| rs16927997 | CC | 4/4 (100.00) | / | / |
|  | CT | 73/94 (77.66) | 1.45 (0.86-2.46) | 1.25 (0.70-2.20) |
|  | TT | 300/427 (70.26) | 0.64 (0.38-1.09) | 0.71 (0.39-1.13) |
| rs430397 | AA | 27/33 (81.82) | 1.83 (0.74-4.52) | 1.74 (0.67-4.18) |
|  | AG | 112/154 (72.73) | 1.07 (0.70-1.62) | 1.05 (0.67-1.55) |
|  | GG | 238/338 (70.41) | 0.82 (0.55-1.23) | 0.83 (0.55-1.25) |
| rs11355458 | GG | 16/30 (53.33) | 0.42 (0.20-0.89) | 0.46 (0.25-0.94) |
|  | dG^§^ | 114/167 (68.26) | 0.78 (0.52-1.16) | 0.81 (0.53-1.19) |
|  | dd^§^ | 247/328 (75.30) | 1.57 (1.07-2.32) | 1.52 (1.03-2.30) |
| rs17840761 | TT | 81/105 (77.14) | 1.41 (0.86-2.33) | 1.35 (0.80-2.27) |
|  | CT | 176/243 (72.43) | 1.06 (0.72-1.55) | 1.05 (0.72-1.54) |
|  | CC | 120/177 (67.80) | 0.75 (0.50-1.11) | 0.79 (0.53-1.15) |
| rs17840762 | TT | 24/33 (72.73) | 1.05 (0.48-2.32) | 1.12 (0.53-2.39) |
|  | CT | 119/162 (73.46) | 1.13 (0.74-1.71) | 1.15 (0.75-1.72) |
|  | CC | 234/330 (70.91) | 0.89 (0.60-1.32) | 0.85 (0.58-1.28) |
| rs391957 | AA | 16/30 (53.33) | 0.42 (0.20-0.89) | 0.46 (0.25-0.94) |
|  | AG | 114/167 (68.26) | 0.78 (0.52-1.16) | 0.81 (0.53-1.19) |
|  | GG | 247/328 (75.30) | 1.57 (1.07-2.32) | 1.52 (1.03-2.30) |
| rs372225 | TT | 57/89 (64.04) | 0.65 (0.40-1.05) | 0.76 (0.47-1.12) |
|  | CT | 212/295 (71.86) | 1.01 (0.69-1.48) | 1.08 (0.74-1.54) |
|  | CC | 108/141 (76.60) | 1.40 (0.90-2.19) | 1.30 (0.81-2.07) |
| rs10819043 | CC | 59/73 (80.82) | 1.78 (0.96-3.29) | 1.54 (0.80-2.79) |
|  | CT | 188/273 (68.86) | 0.74 (0.50-1.08) | 0.85 (0.66-1.16) |
|  | TT | 130/179 (72.63) | 1.06 (0.71-1.59) | 1.05 (0.71-1.59) |
| rs359587 | TT | 117/175 (66.86) | 0.70 (0.47-1.04) | 0.79 (0.55-1.15) |
|  | AT | 178/236 (75.42) | 1.39 (0.94-2.04) | 1.32 (0.85-1.95) |
|  | AA | 78/114 (68.42) | 0.81 (0.52-1.27) | 0.82 (0.53-1.29) |
| rs12202 | CC | 34/40 (85.00) | 2.35 (0.96-5.71) | 2.11 (0.85-4.96) |
|  | CG | 113/160 (70.63) | 0.92 (0.61-1.39) | 0.84 (0.59-1.33) |
|  | GG | 230/325 (70.77) | 0.87 (0.59-1.30) | 0.89 (0.59-1.34) |
| rs7046471 | CC | 37/52 (71.15) | 0.97 (0.51-1.82) | 0.92 (0.49-1.75) |
|  | CT | 160/236 (67.80) | 0.70 (0.48-1.02) | 0.79 (0.55-1.15) |
|  | TT | 180/237 (75.95) | 1.46 (0.99-2.15) | 1.41 (0.96-2.11) |
| rs10986849 | GG | 25/29 (86.21) | 2.56 (0.87-7.48) | 1.90 (0.54-6.52) |
|  | AG | 119/163 (73.01) | 1.09 (0.72-1.65) | 1.13 (0.71-1.67) |
|  | AA | 233/333 (69.97) | 0.78 (0.52-1.16) | 0.82 (0.53-1.17) |
| rs4837022 | CC | 249/341 (73.02) | 1.18 (0.80-1.76) | 1.22 (0.84-1.81) |
|  | CT | 105/157 (66.88) | 0.71 (0.48-1.07) | 0.73 (0.45-1.20) |
|  | TT | 23/27 (85.19) | 2.34 (0.80-6.88) | 1.64 (0.73-5.77) |
| rs10118570 | GG | 7/15 (46.67) | 0.33 (0.12-0.94) | 0.44 (0.20-0.93) |
|  | AG | 105/150 (70.00) | 0.88 (0.58-1.34) | 0.71 (0.48-1.05) |
|  | AA | 265/360 (73.61) | 1.32 (0.88-1.97) | 1.33 (0.88-1.99) |
| rs10760403 | GG | 22/28 (78.57) | 1.47 (0.58-3.69) | 1.31 (0.53-3.25) |
|  | TG | 92/132 (69.70) | 0.87(0.57-1.34) | 0.87 (0.57-1.31) |
|  | TT | 263/365 (72.05) | 1.04 (0.69-1.57) | 1.06 (0.66-1.59) |
| rs1477147 | GG | 27/37 (72.97) | 1.07 (0.50-2.26) | 1.12 (0.52-2.33) |
|  | CG | 120/176 (68.18) | 0.77 (0.52-1.14) | 0.79 (0.55-1.19) |
|  | CC | 230/312 (73.72) | 1.26 (0.86-1.85) | 1.20 (0.80-1.84) |
| rs10819146 | CC | 63/88 (71.59) | 0.99 (0.59-1.64) | 0.94 (0.55-1.60) |
|  | CG | 180/240 (75.00) | 1.34 (0.91-1.97) | 1.32 (0.88-1.95) |
|  | GG | 134/197 (68.02) | 0.74 (0.51-1.10) | 0.81 (0.55-1.15) |
| rs531599 | CC | 29/38 (76.32) | 1.29 (0.59-2.79) | 1.22 (0.54-2.76) |
|  | CG | 125/165 (75.76) | 1.34 (0.88-2.04) | 1.31 (0.86-2.03) |
|  | GG | 223/322 (69.25) | 0.72 (0.48-1.07) | 0.77 (0.53-1.11) |
| rs10760443 | TT | 171/238 (71.85) | 1.00 (0.69-1.47) | 1.05 (0.71-1.49) |
|  | CT | 177/242 (73.14) | 1.13 (0.77-1.66) | 1.15 (0.74-1.67) |
|  | CC | 29/45 (64.44) | 0.69 (0.36-1.31) | 0.59 (0.35-1.29) |
| rs12336217 | AA | 178/234 (76.07) | 1.47 (1.00-2.17) | 1.39 (0.90-1.88) |
|  | AG | 171/249 (68.67) | 0.75 (0.51-1.09) | 0.79 (0.55-1.14) |
|  | GG | 33/42 (78.57) | 1.48 (0.69-3.18) | 1.45 (0.69-3.17) |
| rs3850585 | TT | 69/88 (78.41) | 1.52 (0.88-2.63) | 1.52 (0.79-2.65) |
|  | CT | 163/238 (68.49) | 0.74 (0.51-1.09) | 0.75 (0.51-1.08) |
|  | CC | 145/199 (72.86) | 1.09 (0.73-1.61) | 1.07 (0.71-1.58) |
| rs2454217 | GG | 19/24 (79.17) | 1.52 (0.56-4.14) | 1.67 (0.63-4.37) |
|  | AG | 83/118 (70.34) | 0.91 (0.58-1.43) | 0.93 (0.60-1.47) |
|  | AA | 275/383 (71.80) | 1.00 (0.65-1.53) | 0.95 (0.59-1.50) |
| rs487914 | CC | 19/24 (79.17) | 1.52 (0.56-4.14) | 1.67 (0.63-4.37) |
|  | AC | 87/124 (70.16) | 0.90 (0.58-1.40) | 0.91 (0.58-1.40) |
|  | AA | 271/377 (71.88) | 1.01 (0.66-1.55) | 0.99 (0.65-1.53) |
| rs488039 | GG | 17/20 (85.00) | 2.28 (0.66-7.91) | 1.67 (0.63-4.37) |
|  | AG | 83/119 (69.75) | 0.88 (0.56-1.37) | 0.95 (0.68-1.44) |
|  | AA | 275/386 (71.24) | 0.90 (0.58-1.39) | 0.87 (0.56-1.38) |
| rs1890546 | CC | 29/40 (72.50) | 1.04 (0.50-2.14) | 1.09 (0.53-2.19) |
|  | CT | 137/184 (74.46) | 1.23 (0.82-1.84) | 1.14 (0.79-1.80) |
|  | TT | 211/301 (70.10) | 0.82 (0.56-1.21) | 0.84 (0.56-1.25) |

^*^RR, relative risk; CI, confidence interval.

^†^Calculated using Pearson Chi-square test.

^‡^Adjusted for age, gender, smoking, drinking and HBsAg.

^§^d, the deleted base.


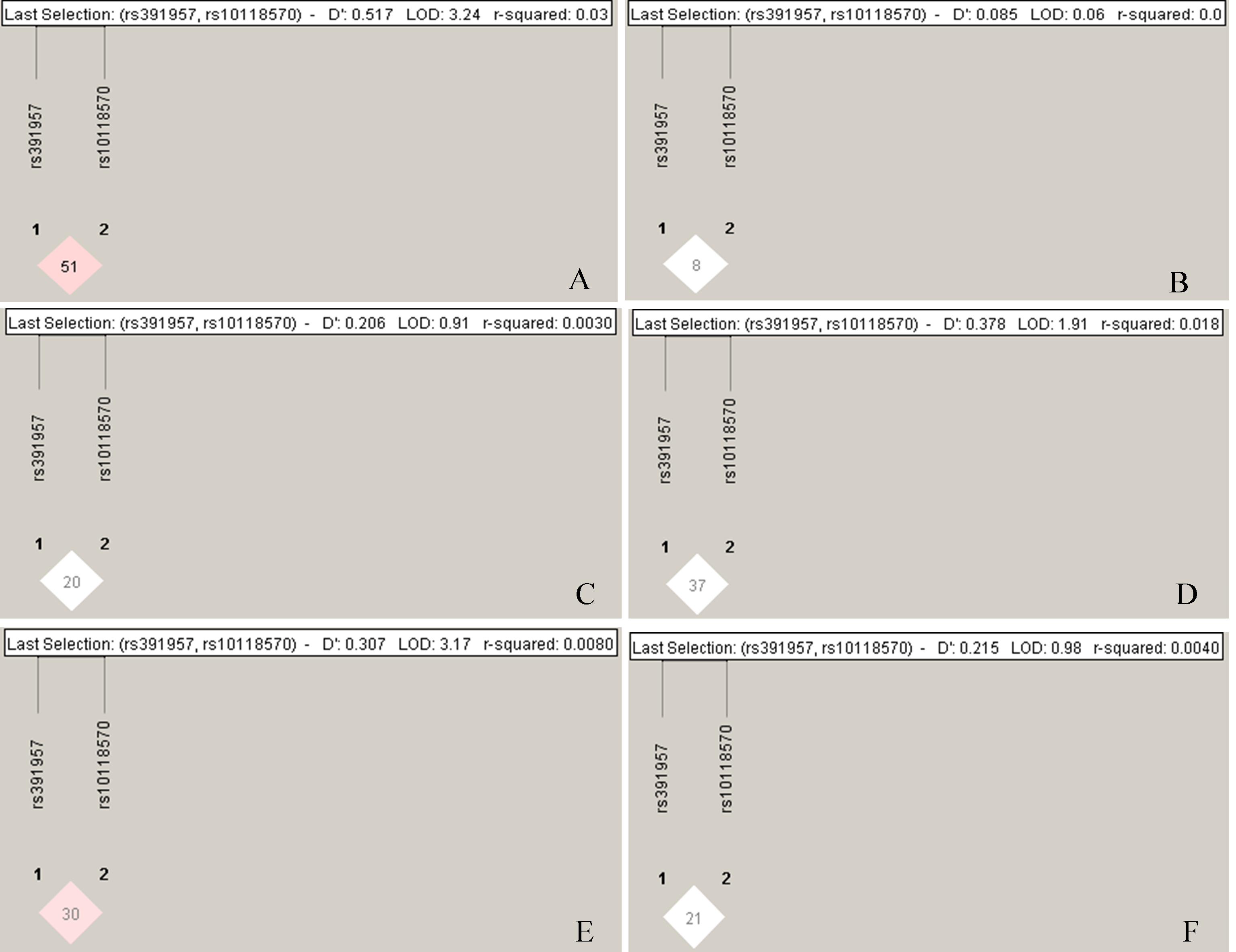


**Figure S1.** Linkage disequilibrium (LD) mapping of rs391957 and rs10118570 showed that these two SNPs were NOT linked. (A) Controls in the discovery cohort. (B) Cases in the discovery cohort. (C) Controls in the replicative cohort. (D) Cases in the replicative cohort. (E) Controls in the combined cohort. (F) Cases in the combined cohort.
